# Coral symbioses under prolonged environmental change: living near tolerance range limits

**DOI:** 10.1038/srep36271

**Published:** 2016-11-02

**Authors:** Eugenia M. Sampayo, Tyrone Ridgway, Lorenzo Franceschinis, George Roff, Ove Hoegh-Guldberg, Sophie Dove

**Affiliations:** 1 School of Biological Sciences, University of Queensland, St. Lucia, QLD 4072, Australia; 2 ARC Centre of Excellence for Coral Reef Studies, University of Queensland, St. Lucia, QLD 4072, Australia; 3 Global Change Institute, University of Queensland, St. Lucia, QLD 4072, Australia

## Abstract

As climate change progresses, understanding the long-term response of corals and their endosymbionts (*Symbiodinium*) to prolonged environmental change is of immediate importance. Here, a total of 1152 fragments from 72 colonies of three common coral species (*Stylophora pistillata*, *Pocillopora damicornis*, *Seriatopora hystrix*) underwent a 32-month reciprocal depth transplantation. Genetic analysis showed that while *S. hystrix* maintained its generalist symbiont, some *S. pistillata* and *P. damicornis* underwent temporary changes in resident symbionts immediately after stress (transplantation; natural bleaching). These temporary changes were phylogenetically constrained to ‘host-compatible’ symbionts only and reversion to original symbionts occurred within 7 to 12 months, indicating long-term fidelity and stability of adult symbioses. Measurements of symbiont photo-physiology (dark adapted yield, pressure over photosystem II) and coral health (host protein, bleaching status, mortality) indicated a broad acclimatory capacity. However, this came at an apparent energetic expense as disproportionate mortality amongst symbioses that persisted outside their distribution range was observed following a natural bleaching event. As environmental changes due to climate change become more continuous in nature, sub-lethal effects linked to the existence near tolerance range limits coupled with the inability of adult coral colonies to change resident symbionts makes corals particularly susceptible to additional environmental fluctuations or stress events and reduces the resilience of coral populations.

The symbiosis between reef-building corals and their endosymbiotic dinoflagellates (*Symbiodinium*) underpins the success of coral reefs. Over the past few decades, coral reefs have suffered global declines. Climate change poses a significant threat to the persistence of reef ecosystems^1^ as increases in sea surface temperatures lead to widespread coral bleaching and associated mortality. The bleaching response is highly variable at the colony level and has, at least in part, been related to the presence of specific resident symbionts^2^,^3^,^4^,^5^. Organismal tolerance limits are the combined outcome of the tolerance limits of the coral host together with its photosynthetic symbionts^6^, and a coral species can occupy a broad environmental range by associating with different symbionts^7^,^8^,^9^,^10^,^11^. The genus *Symbiodinium* contains several major genetic clades, each of which are subdivided into ‘types’ that represent eco-types, phylo-types or species^12^. Given their broad diversity and short generation time, the symbionts represent a likely source of rapid adaption in corals and, considering the pace of climate change, this idea has received much attention over the past decades^2^,^3^,^5^,^13^,^14^,^15^.

Flexibility of established, adult coral symbioses has been a point of debate between scientists for over two decades^16^,^17^,^18^,^19^. Initial suggestions that stress acts as a driver of adaptive change by facilitating displacement of resident sensitive symbionts with tolerant ones^13^ found support in reports that thermally tolerant symbionts spread across coral communities following bleaching events^14^,^16^. Repeated monitoring prior, during and after thermal events, however, provided contrasting evidence that observed community shifts occurred through differential mortality amongst coral colonies harbouring sensitive versus tolerant resident symbionts as opposed to symbiont changes within individual colonies^3^,^4^,^5^. Further work showed that some corals did undergo a shift in resident symbionts but these changes were mostly transient and original symbionts re-established after stress conditions subsided^3^,^4^,^5^,^19^,^20^. Particularly, the stress-induced association with thermally tolerant symbionts (specifically D1a or *Symbiodinium trenchii*) has received much attention because it, albeit temporarily, increases thermal tolerance by 1–2 °C and improves post-stress survival^2^,^3^,^5^. The unstable nature of resident symbiont shifts may be due to the ‘transient’ nature of stress events and the predicted chronic increases in future ocean temperatures may allow heat-tolerant symbionts to persist. Given that physiological trade-offs are linked to specific symbionts^21^,^22^, the pertinent question whether the ecological costs of changing symbionts outweigh the benefits remains.

Few studies have determined long-term flexibility and ecological trade-offs of coral symbioses under prolonged environmental change (maximum of 14 months^23^,^24^,^25^). Given the logistical constraints of field-based temperature manipulations^25^, we achieved prolonged environmental change by altering the light environment of three coral species, *Stylophora pistillata*, *Pocillopora damicornis*, and *Seriatopora hystrix*. These coral species display different levels of light-related symbiont niche partitioning^9^, where *S. hystrix* harbours only a single depth generalist (*Symbiodinium* C3nt) while *S. pistillata* and *P. damicornis* associated with both depth generalists and specialists. A total of 1152 fragments from 72 colonies of the three coral species were reciprocally transplanted for a 32-month period to examine whether permanent replacement of resident dominant symbionts occurred under persistent environmental change, and if bleaching or stress is a prerequisite for modifying symbionts. The aim of this long-term experiment was not to pinpoint the source of symbionts changes (within colony versus external). Instead, dominant resident symbionts are identified and matched with physiological indicators to understand how adult coral symbioses respond, with or without changes in resident symbionts, when pushed to live near or outside currently established tolerance range (niche) limits.

## Methods

### Sample collection, experimental setup and *in situ* temperature and light conditions

**Figure 1:**
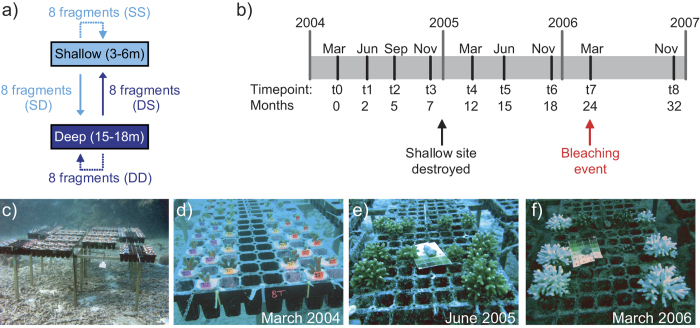
(**a**) Experimental design of reciprocal transplants from shallow to shallow (SS), shallow to deep (SD), deep to deep (DD) and deep to shallow (DS). **(b)** Timeline and sampling points. **(c)** Fragments of control and transplanted colonies fixed into seedling trays and mounted on aluminium frames. The **(d)** small initial coral fragments (March 2004) grew to **(e)** small colonies by June 2005 and **(f)** some were visibly pale during a natural bleaching event in March 2006.

A total of 32 *Stylophora pistillata,* 20 *Pocillopora damicornis* and 20 *Seriatopora hystrix* colonies were collected at Harry’s Bommie, Heron Island (Australia; 23°27.625″S; 151°55.759″E); half of the colonies originated from 3 to 6 meters (shallow) and half from 15 to 18 meters (deep). Conspecific colonies were collected at least 10 meters apart to avoid sampling clonemates. A total of 16 fragments of approximately 4 cm length were removed from the top parts of each colony and fixed into plastic seedling trays with underwater cement (Mapei Granirapid, Australia). Eight of these fragments were returned to their collection depth (controls), while the remaining eight were transplanted to the reciprocal depth. This resulted in four treatments (Fig. 1a): shallow to shallow (control, SS), shallow to deep (SD), deep to deep (control, DD) or deep to shallow (DS). A fragment from each colony and each treatment was removed at each of 8 time-points (Fig. 1b): June, September and November 2004 (t = 1, 2, 3 respectively); March, June and November 2005 (t = 4, 5, 6); March and November 2006 (t = 7, 8). The time points were 2, 5, 7, 12, 15, 18, 24 and 32 months after the start of the experiment (t = 0) in March 2004. Seedling trays were attached in random positions to aluminium frames approximately 60 cm off the bottom near reef structure to prevent sedimentation (Fig. 1c) and photographed at each time point (Fig. 1d–f).

Data loggers were placed *in situ* to continuously record water temperature and irradiance at 30-minute intervals between 2004–2006 (Odyssey, Data Flow Systems, NZ). At each opportunity during this time period, loggers were retrieved, downloaded and returned to the field immediately. Yearly temperature averages were calculated from 30-day running averages but excluded the thermal anomaly between November 2005 and February 2006. A paired t-test was used to test if daily average temperatures differed between shallow and deep. Although the light loggers were in the field continuously, data could only be used for the first 5 days after each deployment to exclude fouling effects. A total of 75 data points over the entire period were obtained and the average light level over a 24-hour period was calculated to obtain the total irradiance available for photosynthesis and exclude effects of seasonality in daylight hours. Regression analysis was done on seasonal irradiance fluctuations by fitting the wave-function, f = y0 + a*sin(2*π*x/365–1.6181), to the raw data.

### Physiological measurements and genetic identification of Symbiodinium

The genetic identity of symbionts at the initial setup of the experiment was established and assumed to represent the stable symbiotic state^4^,^9^,^26^,^27^. Spatial homogeneity within colonies has been shown at the level of individual clonal lines^26^,^27^, and the specific experimental species used here have been shown to contain a single dominant symbiont ITS2-type throughout the colony in the majority of colonies (>98.2%^9^). As such, we assume resident symbionts were similar across all internal colony replicates and temporal changes were not due to initial within-colony differences. Genetic samples were collected from a colony representative sample across treatments at each of the eight sampling points (Fig. 1b), which included a mild bleaching event in early 2006 (t = 7) and eight months post-bleaching (t = 8) (Fig. 1b,f). Unfortunately, a severe storm destroyed the shallow part of the experiment after seven months (t = 3). The deep setup containing deep controls (DD) and shallow transplants (SD) remained intact. To compensate for the loss of the shallow controls to SD transplants, additional samples were collected from random shallow colonies at each time point.

At each sampling time-point, colony pigmentation was visually assessed *in situ* and fragments were photographed for reference (bleached, partially bleached, normal). Survival rate over time was calculated for each colony as the percentage of surviving fragments relative to the previous time-point. One transplant and one control fragment sampled from each colony were placed in shaded flow-through aquaria and the dark-adapted yield of photosystem II (*F*_v_/*F*_m_) was measured 4 hours after sunset using a Diving PAM-fluorometer (Walz, Germany). Coral tissue was then removed using an airgun, the homogenate centrifuged and the symbiont pellet preserved in 20% DMSO preservation buffer (stored at −20 C)^28^. The coral water-soluble protein content was measured from the homogenized tissue slurry supernatant by recording absorbance at 235 and 280 nm (SHIMADZU UV-2450 spectrophotometer). Protein content was calculated^29^ and standardized to fragment surface area with the wax-weight method^30^. *Symbiodinium* types were characterised following standard molecular methods^5^,^9^,^31^,^32^ using denaturing gradient gel electrophoresis (DGGE) fingerprinting of the nuclear ribosomal internal transcribed spacer region 2 (ITS2 rDNA). The symbionts in the three coral species have been described as ecologically and phylogenetically cohesive units equivalent to species using a variety of genetic markers^31^. The ITS2-DGGE methodology accurately identifies resident dominant symbiont species at densities as low as 0.2 to 10% (depending on the species^20^,^31^,^32^,^33^).

In addition to measurements taken at each time point, excitation pressure over photosystem II (Q_m_)^8^ was recorded in November 2005 (t = 6). The effective quantum yield (*F*_q_′/*F*_m_′) was measured *in situ* at noon, after which fragments were collected and transferred to aquaria, where maximum *F*_v_/*F*_m_ (dusk) and dark-adapted *F*_v_/*F*_m_ (4 h after sunset) were measured. Triplicate measurements were taken on different locations of the same fragment (by this time-point grown to the size of a small colony) approximately 1 cm below branch tips.

### Statistical analysis

The time series data for host protein and dark-adapted yield (*F*_v_/*F*_m_) were analysed for each coral species independently using a univariate mixed model permutational analyses of variance (PERMANOVA, Primer-e v6.0) with Euclidian distance, 9999 permutations, Type III error, and permutation of residuals under an unrestricted model^34^. Euclidean distance for univariate analyses produce sums of squares estimates equivalent to parametric ANOVA, but significance can be tested without the assumption of normality^34^. PERMDISP was used to test for homogeneity of dispersion (equivalent to homogeneity of variances) on raw as well as Log(x + 1), ArcSin, and BoxCox transformed data. Transformation did not improve variances (except for the *S. hystrix* protein data set) and, thus, a conservative p-value of 0.01 was used^35^,^36^ on the log-transformed protein or raw *F*_v_/*F*_m_ data for all species. By using a permutation based linear mixed model approach instead of non-parametric statistics we could test for interactive effects between fixed factors (symbiont_treatment; season)^36^,^37^,^38^ and account for sampling separate fragments from individual colonies over time by including ‘individual’ as a random factor. In addition, within colony effects were tested using *F*_v_/*F*_m_ data measured for all (pre-treatment) fragments from the same colony at the start of the experiment (not possible for protein content because the sampling method is destructive). We thus evaluate whether variance between fragments of the same colony (individual) exceeded variance between individuals (at t0) and tested whether individual colony effects exist over time (inclusion of ‘individual’ as a random factor). In both instances ‘individual’ was not significant in either the protein or *F*_v_/*F*_m_ datasets for all coral species and the random factor was excluded from further post-hoc comparisons. Differences in survival over time between treatments and symbiont types were visualized using Kaplan-Meier (KM) log-rank survival analysis with the “survival” R package^39^. A random-effects Cox proportional hazard model was conducted using the “coxme” R package^40^ to analyze differences in survival among symbiont types and treatments, with individual incorporated as a random effect. Data from the more detailed photosynthetic measurements taken at t = 6 (Qm, *F*_q_′/*F*_m_′ and dark-adapted *F*_v_/*F*_m_) conformed to assumptions of normality and homoscedascity, after which ANOVAs were run with ‘symbiont_treatment’ as a single fixed factor (at this time-point, no within treatment ‘colony effects’ were present and all remaining fragments were treated as replicates). Post-hoc significance was tested using a Fishers LSD test (Statistica 6.0, StatSoft, Tulsa, OK, USA).

## Results

### Local temperature and light conditions

**Figure 2:**
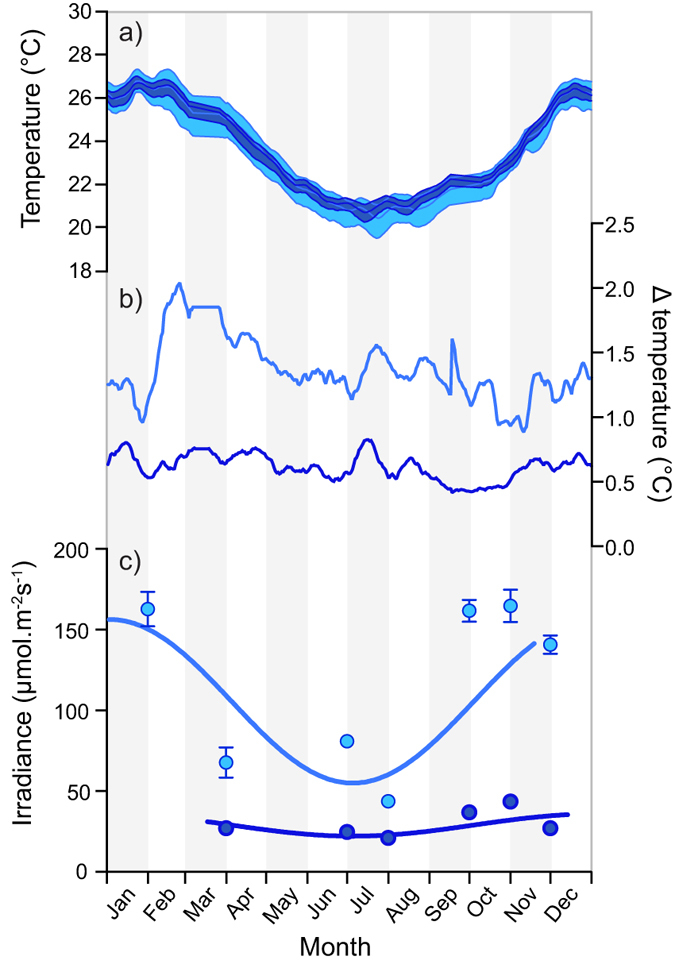
Average yearly **(a)** temperature, **(b)** difference between maximum and minimum temperature and **(c)** overall daily irradiance for the shallow and deep transplantation sites. Light blue = shallow (3 − 6m) and dark blue = deep (15 − 18 m).

The average daily temperature between the deep and shallow site did not differ (*n* = 697, *t* = 1.485, *p *> 0.1; Fig. 2a). However, daily temperature fluctuations (ΔT = T_Max_–T_Min_) in the shallow (ΔT = 1–2 °C) were at least double that of the deep (ΔT = 0.4–0.8 °C, Fig. 2b). Seasonal fluctuations in average daily irradiance were also higher in the shallows (110–314 μmol quanta.m^−2^.s^−1^) compared to the deep (44–65 μmol quanta.m^−2^.s^−1^; Fig. 2c).

### Bleaching status and changes in symbiont communities over time

Significant differences were observed in symbiont communities over time among the three coral species. Specimens of *Seriatopora hystrix* maintained their generalist symbiont C3n-t at both depths throughout the experimental period.

Shallow *Pocillopora damicornis* initially contained symbiont C42a (*n* = 3) or C33a (*n* = 7) whereas deep colonies had C33a (*n* = 9) or C33 (*n* = 1). At t = 1, C33a was detected alongside C33 in the C33 colony irrespective of treatment (DD, DS) and this remained stable throughout the experiment. In two of three shallow C42a colonies, C33a was detected as a secondary symbiont prior to handling (t = 0). Transplantation (SD) caused this secondary C33a to become undetectable at t = 1, but it re-appeared at t = 3 and the mixed profile remained stable for the duration of the experiment. Other than these three colonies, no other colonies underwent symbiont changes at any time during the experiment.

**Figure 3:**
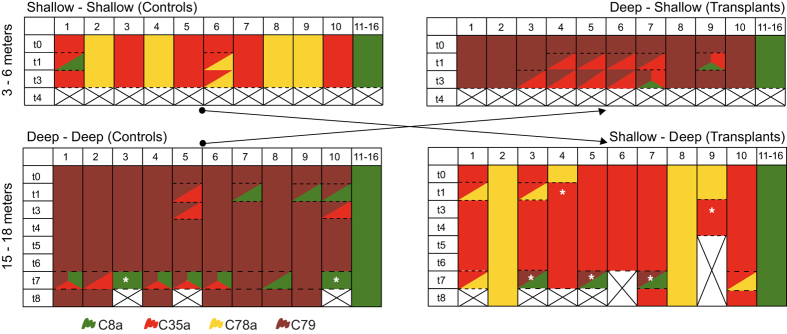
Changes in symbiont types harboured by *Stylophora pistillata* over the 32 month experiment. Colours represent different symbiont types: red = C35a, yellow = C78a, maroon = C79, green = C8a. Mortality between time periods is indicated by crosses, and colonies that lost their original symbiont type are indicated with ‘*’. Partitioned squares indicate symbionts co-occur in a single colony.

*Stylophora pistillata* colonies showed the most pronounced symbiont changes (Fig. 3). At t = 0, deep *S. pistillata* harboured either C79 (n = 10) or C8a (n = 6) whereas shallow colonies had C35a (n = 6), C78a (n = 4), or C8a (n = 6). Colonies with generalist C8a maintained this symbiont throughout the experiment regardless of treatment. The four colonies with shallow-specialist C78a remained stable when kept in the shallows (SS) but when transplanted to the deep (SD), two switched to C35a that was maintained until they died (t = 5, 7). Two of the six controls (SS) with shallow-specialist C35a initially gained either C8a or C78a (t = 1) and these secondary types persisted until the shallow experiment was destroyed (t = 3). Two of the transplanted (SD) C35a colonies initially gained C78a but reverted back to C35a (t = 3). Of the ten deep controls (DD) with deep-specialist C79, four initially gained C35a or C8a but these disappeared after t = 3. Six of the transplanted C79 colonies (DS) also gained C35a or C8a, which remained present until the shallow experiment ended. After initial changes (t = 1 to 3), all symbionts in the deep remained stable until bleaching occurred (t = 7; Fig. 3).

Unlike *P. damicornis* and *S. hystrix*, which never visibly bleached, *S. pistillata* showed significant bleaching but only at t = 7. Bleaching sensitivity amongst colonies was variable and could directly be related to the resident symbiont type. All colonies with C79 or C35a severely bleached irrespective of treatment (DD or SD) while all colonies containing symbiont types C8a or C78a remained visually normally pigmented. These data correspond to field observations of ‘wild’ colonies from a previous study^4^, which showed that colonies were sensitive to bleaching when associating with C79 (100% of wild colonies completely or over 50% of the colony bleached) or C35a (94% of wild colonies bleached) and showed no visible signs of bleaching when harbouring C8a or C78a. Here, this differential bleaching sensitivity was coupled to changes in resident symbionts. Bleaching sensitive colonies with C79 or C35a gained additional symbionts while those with C8a and C78a showed no change during or post-bleaching (Fig. 3). During bleaching (t = 7), one transplanted (SD) C35a colony remained stable while the rest either gained a secondary symbiont (C78a) or completely displaced resident C35a with a combination of C79 and C8a. After the bleaching event, all but two of these colonies that originally contained C35a died (Fig. 3; t = 8). Of the ten deep C79 controls (DD), six gained C35a and/or C8a, two were completely replaced by C8a and two remained stable with C79 during bleaching. Of the eight that underwent changes, five reverted back to C79 and the remaining three died, including those that had adopted the more thermally tolerant C8a.

### Survival analyses

**Figure 4:**
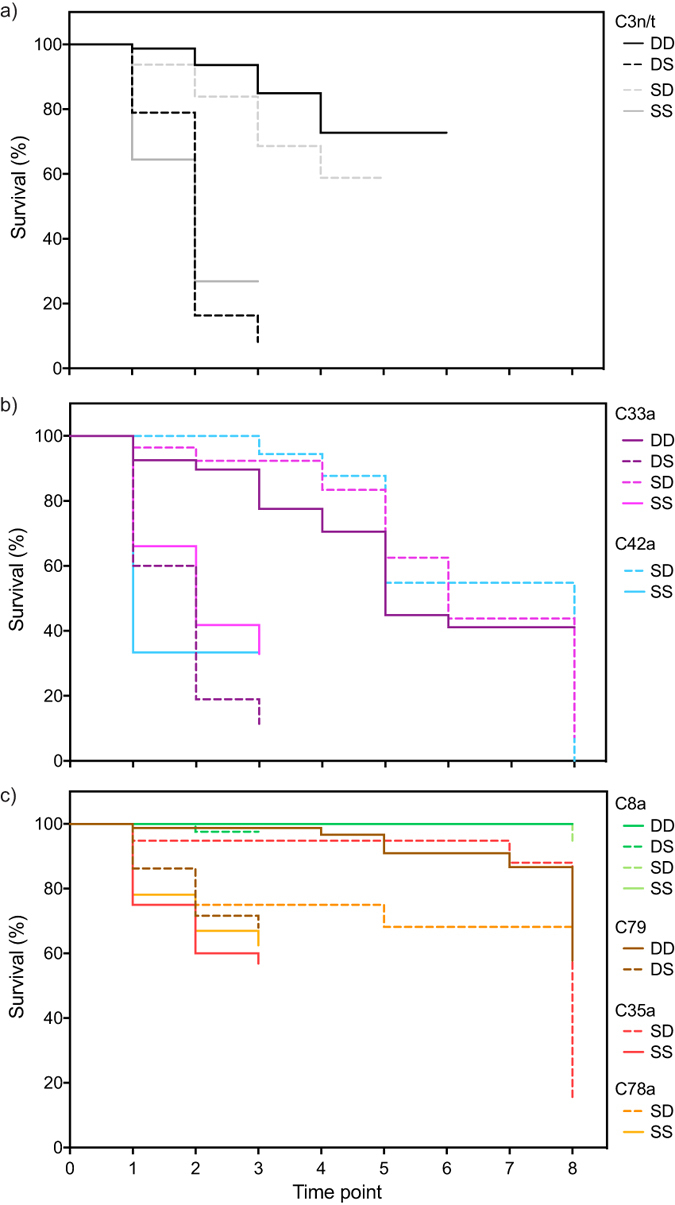
Kaplan Meier survival plots for each ‘symbiont-treatment’ group of **(a)** *Seriatopora hystrix,* **(b)** *Pocillopora damicornis* and **(c)** *Stylophora pistillata*. Treatments were shallow to shallow (SS), shallow to deep (SD), deep to deep (DD) and deep to shallow (DS).

Across species, mortality was highest in the shallows (t = 0 to 3; Fig. 4). In *S. hystrix,* up to t = 3, shallow mortality rates were high irrespective of treatment (Fig. 4a; SS vs. DS no significant difference, Table S1) with only 14–19% of fragments surviving compared to deep controls (DD) or transplants (SD) with 59 and 85% respectively (Fig. 4a, p = 0.001; Table S1). Survival steadily decreased, and after t = 6, too few specimens remained to calculate rates for *S. hystrix*.

For *P. damicornis*, initial survival (up to t = 3) was also comparatively low in the shallows compared to the deep. Shallow specimens, which included C42a controls and C33a controls and transplants (DS), exhibiting significantly lower survival (32, 33, 6% respectively) compared to deep controls (C33a DD; 85%) and shallow to deep transplants (SD C33a or C42a; >90%) (Fig. 4b, SS and DS vs. DD and SD, p = 0.001; Table S1). Importantly, within depth, specimens with C33a that were transplanted from the deep to the shallow experienced significantly higher mortality compared to local shallow controls (DS vs. SS, p = 0.001, Table S1) but transplantation in the opposite direction did not negatively influence survival (C33a SD not significant from DD; Table S1). Up to t = 6, survival rates of deep *P. damicornis* were between 39–56% irrespective of treatment. Although no colonies were visibly bleached at t = 7, significant post-bleaching mortality occurred but only in transplanted (SD) individuals (C33a and C42a). Deep C33a controls (DD) were stable (t = 5 to 8) with no bleaching related mortality (Fig. 4b, Table S1).

Mortality of *S. pistillata* was linked to symbiont type and treatment. Colonies with C8a underwent no mortality from transplantation or bleaching (Fig. 4c; Table S1). Controls with shallow-specialists C35a (SS) exhibited significantly higher mortality than transplants to the deep (SD) at t = 3 (Fig. 4c, p = 0.027; Table S1), while no significant differences were observed among between shallow controls (SS) and shallow transplants (SD) for C78a (Fig. 4c; Table S1). Transplanted deep-specialist C79 (DS) exhibited significantly lower survival than controls (DD, Fig. 4c, p = 0.001; Table S1). In comparison to the other coral species, initial post-transplant survival (t = 1 to 3) in *S. pistillata* was relatively high with overall 59–100% survival in the shallows and 78–100% in the deep. Survival remained largely stable up to t = 7, after which 100% *S. pistillata* with symbionts C79 or C35a were visibly bleached and experienced high post-bleaching mortality. Deep C79 controls (DD) and transplanted (SD) C35a experienced 56 and 97% post-bleaching mortality respectively whereas those with C78a or C8a neither bleached nor died.

### Host protein content

**Figure 5:**
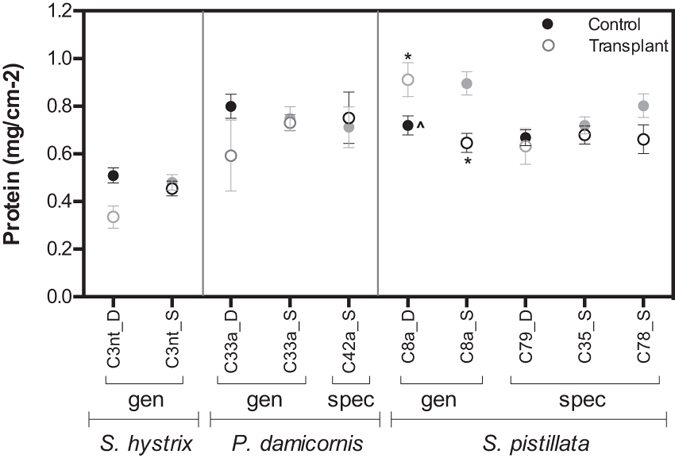
Mean (± SE) host protein content for each ‘symbiont-treatment’ group of the coral species *Seriatopora hystrix, Pocillopora damicornis*, and *Stylophora pistillata.* Depth is indicated with ‘D’ (deep, black symbols) or ‘S’ (shallow, grey symbols), where filled symbols represent controls and open symbols represent transplanted samples (i.e. shallow controls are grey filled, and shallow to deep transplants are black open circles). Significant differences at p ≤ 0.01 are indicated with ‘*’ between controls and transplanted samples (SS and SD or DD and DS). Differences within a single symbiont are indicated with ‘^’ between controls at each depth (SS and DD).

No significant effects were found for ‘season’ or ‘symbiont_treatment’ in the host protein content of *P. damicornis* or *S. hystrix* (Table S2). In these two corals, protein content was similar across months, between depth controls (SS vs. DD) and no significant effect of transplantation was detected. In contrast, *S. pistillata* host protein content was dependant on ‘symbiont_treatment’ (pseudo-F = 7.744, p = 0.001; Table S2) and differed based on the presence of generalist (C8a) or specialist symbionts (C79, C35a, C78a) (Fig. 5). Control specimens with C8a had less protein in the deep than in the shallow (p ≤ 0.006, ‘^’ in Fig. 5; Table S3) and transplantation resulted in a reciprocal change in protein content. In contrast, no differences in protein content were present between controls with specialist symbionts (C79_DD, C35a, SS and C78a_SS). Transplantation did not result in significant changes compared to controls at the original depth (Table S3). When comparing between generalist and specialist symbionts (Fig. 5), deep controls with C8a were similar to deep C79 controls but shallow controls with C8a had higher protein content than shallow controls with C35a and local C79 transplants (DS) (p = 0.007 and p = 0.003; Table S3).

### Dark-adapted photosynthetic yield (F_v_/F_m_)

**Figure 6:**
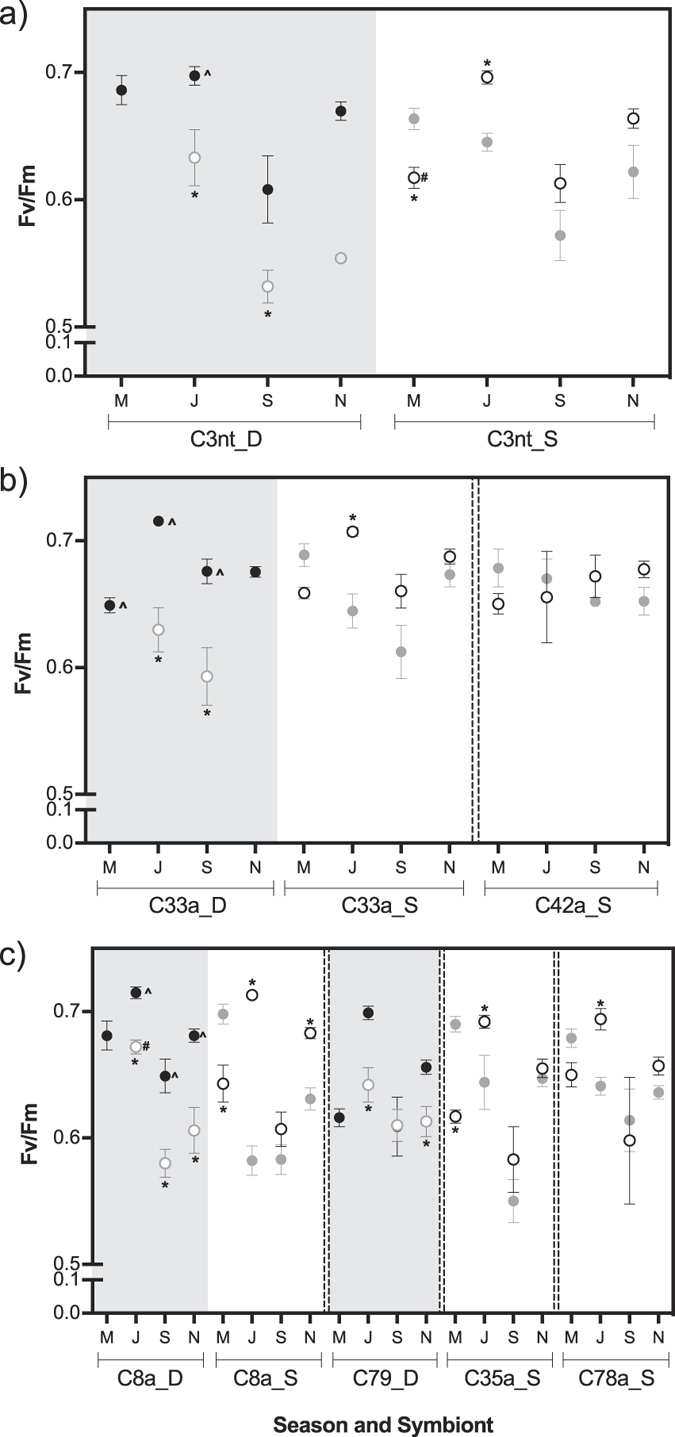
Symbiont dark-adapted photosynthetic yield (*F*_v_/*F*_m_) by season for each ‘symbiont-treatment’ group of the three coral species **(a)** *Seriatopora hystrix,* **(b)** *Pocillopora damicornis*, and **(c)** *Stylophora pistillata.* Originating depth is indicated with ‘D’ (deep) or ‘S’ (shallow). Deep to shallow transplants were absent from the ‘March’ data (see methods). Depth is indicated with ‘D’ (deep, black symbols) or ‘S’ (shallow, grey symbols), where filled symbols represent controls and open symbols represent transplanted samples (i.e. shallow controls are grey filled, and shallow to deep transplants are black open circles). Significant differences at p ≤ 0.01 are indicated with ‘*’ between controls and transplanted samples (SS and SD or DD and DS). Differences within a single symbiont are indicated with ‘^’ between controls at each depth (SS and DD), and with ‘#’ between transplants and post-transplantation depth local controls (SD and DD or DS and SS).

*Symbiodinium* dark-adapted *F*_v_/*F*_m_ showed a significant interaction between ‘season’ and ‘symbiont_treatment’ for all species (*S. hystrix*, pseudo-F = 3.970, p = 0.002; *P. damicornis,* pseudo-F = 5.113, p = 0.001; *S. pistillata*, pseudo-F = 6.014, p = 0.001; Table S4). Irrespective of coral species, symbiont or depth, the lowest *F*_v_/*F*_m_ values were recorded in September (Fig. 6a,c; Table S5 for p-values). However, in March and June values were contrasting between depths. In the shallows, March had the highest *F*_v_/*F*_m_ whereas in the deep, June showed highest seasonal values. Overall, *P. damicornis* with C42a was an exception and showed no differences in *F*_v_/*F*_m_ across months (Fig. 6b, Table S5).

Transplantation led to a similar response across coral species with generalist symbionts, C3nt in *S. hystrix*, C33a in *P. damicornis*, and C8a in *S. pistillata.* Overall, *F*_v_/*F*_m_ of deep controls was higher than shallow controls (Fig. 6a–c, DD vs. SS with p < 0.01 indicated as ^; Table S5). Transplantation led to a reciprocal shift in *F*_v_/*F*_m_ to match values of controls at the transplantation depth. Transplants achieved *F*_v_/*F*_m_ values of local controls in all but two cases, i.e. shallow to deep transplants of *S. hystrix* with C3nt decreased below that of deep controls in March (SD < DD, indicated with # in Fig. 6a) and deep to shallow transplants of *S. pistillata* with C8a had a higher *F*_v_/*F*_m_ compared shallow controls in June (DS > SS; Fig. 6c).

While *P. damicornis* with specialist C42a showed no changes in *F*_v_/*F*_m_ after transplantation, *S. pistillata* colonies with specialist symbionts C79 (deep), and C35a or C78a (shallow) showed a similar transplantation response in *F*_v_/*F*_m_ to corals with generalist symbionts. Despite not occurring at reciprocal transplantation depths, deep specialists became similar to shallow controls with other specialists after transplantation and vice versa. Specifically, when deep C79 were transplanted to the shallows, their *F*_v_/*F*_m_ became similar to values of shallow controls with C78a or C35a (Fig. 6c; Table S5). Similarly, transplanted colonies with shallow C35a or C78a adjusted *F*_v_/*F*_m_ to levels of deep controls with C79 (C78a_SD or C35a_SD vs. C79_DD comparisons not significant; Table S5). However, transplants with specialist C35a differed from local deep controls with generalist C8a (C35_SD vs. C8a_DD, p ≤ 0.01 in March, June, November; Table S5). Transplants with C78a (SD) or with C79 (DS) did not differ from local controls with C8a (DD and SS respectively).

### Pressure over photosystem II (Q_m_)

**Figure 7:**
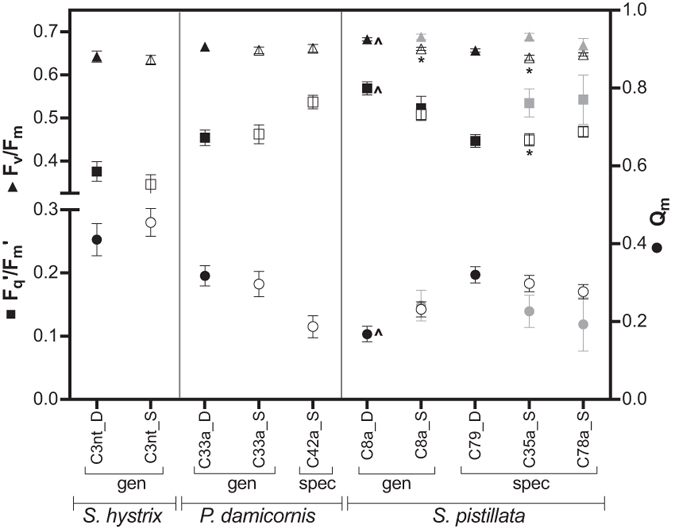
Detailed photosynthetic performance measured in November 2005 only: mean effective quantum yield (*F*_q_′/*F*_m_′; squares, left y-axis), dark-adapted yield (*F*_v_/*F*_m_; triangles, left y-axis) and pressure over photosystem II (Q_m_; circles, right y-axis). Depth is indicated with ‘D’ (deep, black symbols) or ‘S’ (shallow, grey symbols), where filled symbols represent controls and open symbols represent transplanted samples (i.e. shallow controls are grey filled, and shallow to deep transplants are black open circles). Significant differences at p ≤ 0.01 between control and transplants are indicated with ‘*’ and between depth controls (DD and SS) within a symbiont with ‘^’.

The Q_m_ measurements during November 2005 (t = 6) showed no significant effects of ‘symbiont_treatment’ in *S. hystrix*, which had the overall highest Q_m_ across species (Fig. 7). In *P. damicornis*, Q_m_ was significant (F = 3.283, p = 0.049; Table S6) and shallow transplants with C33a (SD) had Q_m_ values similar to deep C33a controls while transplants with shallow-specialist C42a had a significantly lower Q_m_ (Fig. 7, p = 0.04, Table S7). In *S. pistillata* significant effects of ‘symbiont_treatment’ were present on Q_m_, *F*_q_′/*F*_m_′ and *F*_v_/*F*_m_ (F = 5.064, 6.935, 11.592 respectively and all p < 0.001, Table S6). Deep controls with C8a had the lowest Q_m_ compared to other deep samples (irrespective of treatment and symbiont; p < 0.05 for C8a_DD vs. C8a_SD, C79_DD, C35a_SD, C78_SD), but were similar to shallow controls irrespective of symbiont (C8a_SS, C35a_SS, C78a_SS; Fig. 7, Table S7). This trend was mirrored in *F*_q_′/*F*_m_′ and *F*_v_/*F*_m_, where deep C8a controls had significantly higher values compared to all other deep samples (Fig. 7). No differences in Q_m_ were found between shallow specimens, irrespective of treatment or symbiont. Deep controls with specialist symbiont C79 had a significantly higher Q_m_ compared to shallow controls with C35a or C78a but were similar to locally present transplants (SD) with C35a and C78a (Fig. 7).

## Discussion

Our aim was to understand how adult coral symbioses respond, with or without changes in resident symbionts, when pushed to live near or outside established tolerance range limits. Considering the constraints of field-based temperature manipulations, we used long-term changes in irradiance (depth transplantation) to achieve prolonged environmental change (32-months). Increases in irradiance can trigger the same photochemical and cellular response as temperature stress, with examples demonstrating that a transition to an elevated light field can result in PSII photoinactivation and bleaching^10^,^41^,^42^. The potential synergies between light and thermal stress, coupled with the occurrence of a natural thermal stress event during the study, allows us to place our data in the context of understanding how long-term environmental change and punctuated stress events affect coral symbioses. We postulate the expected responses to long-term changes in the environment as: transplanted corals (1) change their symbionts to cope with changed conditions, (2) change symbionts and die, (3) maintain original symbionts and acclimate, or (4) die.

Despite some initial changes in resident symbionts (hypothesis 1), long-term stability of the symbiosis was seen throughout the experiment (hypothesis 3). This result was particularly surprising for *Stylophora pistillata* and *Pocillopora damicornis* because they maintained depth-specialists^9^ even if symbionts better suited (i.e. naturally more common) to the transplantation depth were detected alongside the original symbionts. Generally, corals with generalist symbionts adjusted their host protein content and symbiont photosynthetic performance towards levels of controls at reciprocal transplantation depths. Corals with specialist symbionts also showed an, albeit reduced, acclimatory capacity but without changing their symbionts (hypothesis 3; but two exceptions in *S. pistillata*, Fig. 3). When corals were exposed to additional stress in the form of a thermal bleaching event, transplanted individuals suffered disproportionally higher mortality compared to controls (hypothesis 4), indicating that corals with sub-optimal symbionts were living near limits of their tolerance range (acclimatory potential). Evidently, the long-term response of corals to prolonged environmental change is complex and constitutes components of each of our postulated responses, which differ on temporal scales and are dependant on environmental stability.

### Long-term dynamics in coral symbioses under persistent environmental change

Shallow environments were characterised by an overall higher light environment and larger environmental heterogeneity (temperature and irradiance), making transplantation from deep to shallow likely more stressful than the reverse^8^. While mortality rates and the photosynthetic response corroborated this, it was not reflected in the capacity for changes in resident symbionts as controls and transplants underwent similar levels of temporary change irrespective of the directionality of transplantation.

None of the coral species formed stable novel symbioses in response to persistent environmental change. Irrespective of whether the host was monomorphic or polymorphic, only colonies with specialist symbionts experienced changes in resident symbionts, while those with generalist symbiont types did not. Fluctuations in resident symbionts also occurred in controls (19%) but were more pronounced in transplanted colonies (31%). Changes in symbionts were more pronounced in *S. pistillata* than in *P. damicornis*, where changes were only observed in colonies that already contained mixes (C42a and C33a). This may be related to the extent of polymorphism^9^ or inherent differences in sensitivity between coral and/or symbiont species.

Contrary to the suggestion that bleaching facilitates changes in resident symbionts^13^,^14^,^16^,^43^, our results indicate that visible bleaching is not a prerequisite and disruption of the symbiosis occurred in response to generalised stressors. Colonies with depth-specialists initially gained additional symbiont types but changes were unstable (with the exception of two individuals) and post-stress reversion occurred within seven to twelve months. The corals studied here all vertically transmit the maternal symbionts directly to their offspring. While such symbiosis generally exhibit high levels of specificity^44^,^45^, in horizontally transmitting corals that acquire symbionts from the environment as young recruits, post-stress reversion has also been documented within months^2^,^4^ to years^20^. Based on these time frames, previous studies documenting permanent symbiont changes due to bleaching or transplantation (<12 months^16^,^24^) were potentially terminated before post-stress reversion occurred. Although short-lived increased SST associated with bleaching events likely stimulate post-bleaching reversion, our results show that, even under persistent (irradiance) environmental change, established symbioses are inflexible and highly stable over time.

### Physiological responses to persistent environmental change

Persistence of original symbionts outside their normal environmental range did not have major consequences for the host under stable environmental conditions. Although symbiont density and chlorophyll were not measured, both transplanted and control corals appeared visually healthy throughout the experiment, with host-protein content and symbiont photo-physiology showing remarkable plasticity.

Differences were observed in the ability of depth-specialists versus generalist symbionts to adjust to changes in the light environment. Generalists such as C8a in *S. pistillata,* C33a in *P. damicornis* and C3nt in *S. hystrix*, follow observations that dark-adapted *F*_v_/*F*_m_ is higher in the deep compared to the shallows due to reduced photoinhibition and/or damage at PSII^10^. Transplanted corals with generalist symbionts adjusted dark-adapted *F*_v_/*F*_m_ and host protein content towards values of local controls (i.e. shallow to deep transplants become similar to deep controls and vice versa). In deep to shallow transplants, however, dark-adapted *F*_v_/*F*_m_ fell below values of shallow controls and is potentially indicative of photoinhibition^8^,^10^. While changes in *F*_v_/*F*_m_ of *P. damicornis* and *S. hystrix* were matched by shifts in host protein content (i.e. if *F*_v_/*F*_m_ drops, protein content drops), this pattern was reversed in *S. pistillata*, where a reduction of *F*_v_/*F*_m_ was linked to an increase in protein content. Reductions in *F*_v_/*F*_m_ combined with increases in biomass have previously been observed for unicellular cultures and were explained by the coupling of inactive with active reaction centres (RC). Inactive RC transfer a large proportion of acquired excess light energy to heat, whilst still permitting a portion to go to photochemical quenching and carbon fixation via the active RC^46^. The physiological differences observed here amongst taxa could then point either to intrinsic differences amongst generalist symbionts, or to inter-specific differences in the ability to utilize photo-assimilates, switch to heterotrophy or capitalize on dissolved or particulate organic matter that may be differentially available between depths, when access to photo-assimilates is potentially limited^47^,^48^,^49^.

Pinpointing a general response in corals with specialist symbiont types was more complex as acclimation differed between these symbionts. No significant changes occurred in photosynthetic yield and protein content of *P. damicornis* with shallow C42a, while *S. pistillata* with shallow-specialists C35a and C78a exhibited changes in dark-adapted *F*_v_/*F*_m_ after transplantation and adjusted protein content to levels similar of deep controls with C79. Interestingly, dark-adapted *F*_v_/*F*_m_ of deep-specialist C79 decreased significantly when transplanted to the shallows (indicative of excess light causing photoinhibition), but values were similar to shallow controls with specialists C35a or C78a (Fig. 7c). Controls of shallow and deep specialists were similar. These data show that a single host can achieve comparable photosynthetic yields across its depth range by utilizing different depth specialist symbionts but these have a broad acclimatory potential when transplanted outside of their natural distribution range.

Pressure over photosystem II (Q_m_) has been used to explain depth ranges occupied by a single coral species^8^,^11^. Here, *S. pistillata* with generalist C8a followed patterns of a relative decrease in Q_m_ with depth^8^,^10^. Without reciprocal deep to shallow transplant representatives of deep-specialist C79 (highest Q_m_–high light stress) or control shallow-specialist C42a (low Q_m_–light limitation) it is difficult to speculate how Q_m_ limits the distribution of specialist symbionts. Here, all Q_m_ values were near values indicative of photo-limitation (<0.25^8^), highlighting that this relative measure may not be comparable between species^10^. While being a good indicator of photosynthetic performance, the measured photosynthetic parameters (*F*_v_/*F*_m_, Q_m_) do not necessarily correspond to translocation or carbon production. Symbioses with similar photosynthetic performance can have markedly different translocation of assimilates and can be linked to differences in host proteins, growth or survival^48^.

### Living on the edge: survival and the effects of additional stress

The onset of a thermal stress and subsequent coral bleaching in early 2006 made it apparent that the persistence of out-of-range symbionts came at an overall cost. Colonies maintaining symbionts not normally found in transplantation environments suffered relatively higher mortality compared to those with naturally occurring symbionts. Transplanted *P. damicornis* colonies, while not showing any signs of bleaching, experienced higher mortality compared to controls at the transplantation depth. Similar patterns were seen in transplants of *S. pistillata* with bleaching sensitive symbiont C35a, which experienced approximately 3-fold higher than expected mortality (71%) compared to findings from a field based study documenting post-bleaching mortality of this symbiosis^4^ in its natural shallow environment (19%). Colonies with deep-specialist C79 also suffered high mortality rates (43%) but values were identical to naturally occurring deep colonies (also 43% post-bleaching mortality)^4^, confirming that experimental controls respond similar to ‘wild’ colonies. The disproportionally high mortality amongst transplanted individuals indicates that sub-optimal symbiosis are no longer sustainable at the onset of additional stress and living outside the optimal adaptive or acclimatory state likely comes at a high energetic cost as the host counterbalances the disadvantageous state of its symbiotic component^6^.

### Persistence and adaption of coral symbiosis under future climate change

Symbionts may confer distinct competitive benefits at any life stage^22^,^50^. The observed niche partitioning of symbionts with depth^9^ coupled with temporal stability^4^,^19^,^20^ and broad acclimatory capacity suggests that distinct symbionts are likely advantageous during the early life stages. The coral species in this study vertically transmit symbionts, thereby favouring the persistence of favourable symbionts and high levels of specificity^12^. This evolutionarily defined specificity extends to the temporary changes in resident symbionts observed here, which were restricted to symbionts found within the local conspecifics only. Interestingly, a previous study that documented the rare occurrence of punctuated horizontal symbiont acquisition in vertically transmitting species^51^ showed that this was also limited to symbionts present in local conspecifics. Further findings that juvenile cnidarians with a horizontal transmission strategy preferentially uptake the adult symbiont type^50^,^52^ suggest that symbiont changes may be phylogenetically constrained at the juvenile and adult life stages (see also^19^).

Despite persistently altered environmental conditions and the temporary presence of ‘phylogenetically compatible’ symbionts more suited to transplantation depths, adult symbiosis could not change permanently. Others studies have shown that temporal stability of resident symbionts is high and clonal lines persist within single host colonies (both in vertical, e.g. *Pocillopora* sp.^26^, and horizontal transmitters, e.g. *Acropora* sp.^27^). Strict specificity at the colony level during the adult life stages is congruent with the establishment of host-symbiont recognition pathways during the early life stages^19^. For sessile organisms, benthic establishment during the early life stages defines environmental conditions for life. After locally suitable symbionts are acquired, maintaining their dominance and stability during the adult life stages is likely of greater evolutionary benefit than continued flexibility.

The tolerance range of existing host-symbiont partnerships will play a major role in determining the success of coral species as climate change progresses. Corals with generalist symbionts are expected to have an ecological advantage as they already cope with broad environmental gradients. However, colony level specificity is likely to maintain sub-optimal symbionts under unfavourable conditions, making established corals more vulnerable to ‘add-on’ stress. These processes are expected to dramatically change community composition with reefs ‘adapting’ to thermal stress through the differential mortality of susceptible genotypes and/or species^4^,^5^. Reduced genotypic diversity of both symbiotic partners and the availability of symbiosis with competitive advantages other than thermal tolerance (at any time during the life history), will limit the capacity to cope with further environmental fluctuations. In addition, the transient nature of changes in symbiotic partnerships further indicates little potential to facilitate adaptation in adult corals. Generational turnover of symbionts (mainly horizontal transmitters) or adaptation arising from the coral itself appear more promising^15^,^19^. Generational change represent a slower but more stable adaptive process, in that it leads to the formation of novel non-transient holobionts that are better adapted to a changed environment, and is subsequently less likely to result in substantial losses of genotypic diversity with a lower chance of extinction. The immediate need exists to understand the ecological relevance of these alternative processes and whether these mechanisms would allow corals to keep up with rates of projected global change.

## Additional Information

**How to cite this article:** Sampayo, E. M. *et al.* Coral symbioses under prolonged environmental change: living near tolerance range limits. *Sci. Rep.* **6**, 36271; doi: 10.1038/srep36271 (2016).

**Publisher’s note:** Springer Nature remains neutral with regard to jurisdictional claims in published maps and institutional affiliations.

## Supplementary Material

Supplementary Information
