# Supplementary material for: Coral symbioses under prolonged environmental change: living near tolerance range limits
Source: Sci Rep. 2016 Nov 2;6:36271. doi: 10.1038/srep36271 (PMC5090243; doi:10.1038/srep36271)
Supplement: Supplementary Information [file srep36271-s1.pdf]

Coral symbioses under prolonged environmental change: living near tolerance range limits

Eugenia M. Sampayo*, Tyrone Ridgway, Lorenzo Franceschinis, George Roff, Ove Hoegh-Guldberg, Sophie Dove

*Corresponding Author: e.sampayo@uq.edu.au

Supplementary Tables and Figures

Table S1. Pairwise comparisons from random-effects Cox proportional hazard models for symbiont type and treatment. Significant p-values < 0.05 are shown in bold.

Table S2. Results of a permutation based generalised linear mixed model (PERMANOVA). Analyses were run the 32-month protein dataset for each coral species independently with 'Season' and 'Symbiont_Treatment' as fixed factors and 'Individual' as a random effect. Factors that were significant using a conservative $p \leq 0.01$ (see methods – data analyses) are highlighted in bold.

Table S3. Pairwise comparisons with p-values ≤ 0.01 for significant main effects of the host protein analysis (Table S2).

Table S4. Results of a permutation based generalised linear mixed model (PERMANOVA). Analyses were run on the 32-month dark-adapted photosynthetic yield (F_v/F_m) dataset for each coral species independently with 'Season' and 'Symbiont_Treatment' as fixed factors and 'Individual' as a random effect. Factors that were significant using a conservative $p \leq 0.01$ (see methods – data analyses) are highlighted in bold.

Table S5. Pairwise comparisons with p-values ≤ 0.01 for significant main effects of the dark-adapted F_v/F_m analysis (Table S4).

Table S6. ANOVA results for photosynthetic parameters measured in November 2005on remaining coral fragments. For each species, dark-adapted yield (F_v/F_m), effective yield (F_q'/F_m'), and pressure over photosystem II (Qm) were analysed independently against fixed factor 'Symbiont_Treatment'. Main effects significant at $p < 0.05$ are highlighted in bold.

Table S7. Significant Tukey HSD post-hoc tests for independent single-factorial ANOVAs of the fixed factor 'Symbiont_Treatment' (Table S6) for dark adapted yield (F_v/F_m), effective yield (F_q'/F_m'), and pressure over photosystem II (Qm) measured in November 2005. Significant pairwise comparisons with $p < 0.05$ are shown.

Table S1. Pairwise comparisons from Cox proportional hazard models.

Stylophora pistillata											
		C35a		C78a		C79		C8a			
		SD	SS	SD	SS	DD	DS	DD	DS	SD	SS
C35a	SD	-	**0.027**	0.600	0.110	0.088	0.070	**0.004**	0.170	**0.001**	0.140
	SS	-	-	0.130	0.790	**0.001**	0.460	**0.001**	**0.006**	**0.001**	**0.004**
C78a	SD	-	-	-	0.330	0.058	0.320	**0.003**	0.110	**0.001**	0.093
	SS	-	-	-	-	**0.003**	0.750	**0.000**	**0.016**	**0.001**	**0.013**
C79	DD	-	-	-	-	-	**0.001**	0.058	0.660	**0.025**	0.600
	DS	-	-	-	-	-	-	**0.001**	**0.014**	**0.001**	**0.011**
C8a	DD	-	-	-	-	-	-	-	0.280	0.790	0.310
	DS	-	-	-	-	-	-	-	-	0.180	0.950
	SD	-	-	-	-	-	-	-	-	-	0.200
	SS	-	-	-	-	-	-	-	-	-	-

Pocillopora damicornis							
		C33a				C42a	
		DD	DS	SD	SS	SD	SS
C33a	DD	-	**0.001**	0.460	**0.001**	0.400	**0.001**
	DS	-	-	**0.001**	0.310	**0.001**	0.790
	SD	-	-	-	**0.001**	0.76	**0.001**
	SS	-	-	-	-	**0.001**	0.330
C42a	SD	-	-	-	-	-	**0.001**
	SS	-	-	-	-	-	-

Seriatopora hystrix					
		C3/nt			
		SS	DD	DS	SD
C3n/t	SS	-	**0.001**	0.950	**0.001**
	DD	-	-	**0.001**	0.130
	DS	-	-	-	**0.001**
	SD	-	-	-	-

Table S2. Statistics results host protein content.

Protein data	Factor	df	SS	MS	Pseudo-F	P (perm)
Seriatopora hystrix						
	Ind	34	0.725	0.021	1.562	0.061
	Season	2	0.011	0.006	0.411	0.648
	Symbiont_Treatment	2	0.031	0.015	1.133	0.316
	Season x Symbiont_Treatment	5	0.218	0.044	3.191	0.013
	Res	78	1.064	0.014		
	Total	122	2.213			
Pocillopora damicornis						
	Ind	22	0.794	0.036	1.171	0.306
	Season	2	0.017	0.006	0.189	0.839
	Symbiont_Treatment	5	0.195	0.039	1.265	0.267
	Season x Symbiont_Treatment	9	0.405	0.045	1.459	0.171
	Res	110	3.392	0.031		
	Total	148	4.763			
Stylophora pistillata						
	Ind	75	2.101	0.028	1.313	0.058
	Season	2	0.063	0.031	1.474	0.235
	**Symbiont_Treatment**	5	0.826	0.165	7.744	**0.001**
	Season x Symbiont_Treatment	16	0.614	0.038	1.800	0.031
	Res	254	5.417	0.021		
	Total	356	8.924			

Table S3. Pairwise comparisons (p ≤ 0.01) within significant main effects for host protein.

Protein – Sign. Main Effects		
Seriatopora hystrix (*p* ≤ 0.01)	**Within level**	**p**
no significant main effects		
Pocillopora damicornis		
no significant main effects		
Stylophora pistillata (*p* ≤ 0.01)		
**Symbiont_Treatment**	C8a_DD - C8a_SS, C8a_DS	0.006, 0.006
	C8a_SS - C8a_SD, C79_DD, C79_DS, C35_SS, C35_SD, C78_SD	0.001, 0.002, 0.003, 0.007, 0.003, 0.007
	C8a_DS - C8a_SD, C79_DD, C35_SD	0.001, 0.001, 0.008

Table S4. Statistics results of symbiont dark-adapted photosynthetic yield (F_v/F_m).

Fv/Fm data	Factor	df	SS	MS	Pseudo-F	P (perm)
Seriatopora hystrix						
	Ind	32	0.084	0.003	1.462	0.10
	Season	3	0.128	0.043	23.707	0.001
	Symbiont_Treatment	2	0.070	0.035	19.427	0.001
	**Season x Symbiont_Treatment**	8	0.057	0.007	3.970	**0.002**
	Res	110	0.198	0.002		
	Total	156	0.529			
Pocillopora damicornis						
	Ind	31	0.042	0.001	1.650	0.053
	Season	3	0.006	0.002	2.604	0.065
	Symbiont_Treatment	3	0.054	0.018	21.865	0.001
	**Season x Symbiont_Treatment**	13	0.054	0.004	5.114	**0.001**
	Res	128	0.105	0.001		
	Total	180	0.296			
Stylophora pistillata						
	Ind	63	0.086	0.001	1.268	0.132
	Season	3	0.160	0.053	49.339	0.001
	Symbiont_Treatment	6	0.080	0.013	12.317	0.001
	**Season x Symbiont_Treatment**	25	0.163	0.007	6.014	**0.001**
	Res	309	0.334	0.001		
	Total	409	0.983			

Table S5. Pairwise comparisons ($p \leq 0.01$) of dark-adapted photosynthetic yield (F_v/F_m).

Fv/Fm – Sign. Main Effects			
Seriatopora hystrix ($p \leq 0.05$)	**Within level**	**Within level**	**p**
**Season x Symbiont_Treatment**	Mar	C3nt_SD - C3nt_SS, C3nt_DD	0.003, 0.001
	Jun	C3nt_SS - C3nt_DD, SD	0.001, 0.001
		C3nt_DD - C3nt_DS	0.002
		C3nt_DS - C3nt_SD	0.001
	Sep	C3nt_DS - C3nt_SD	0.006
	C3nt_DD	Sep - Mar, Jun, Nov	0.004, 0.001, 0.007
	C3nt_SS	Sep - Mar, Jun	0.001, 0.001
	C3nt_SD	Jun – Mar, Sep, Nov	0.001, 0.001, 0.004
		Nov – Mar, Sep	0.002, 0.003
Pocillopora damicornis ($p \leq 0.01$)			
**Season x Symbiont_Treatment**	Mar	C33a_SS - C33a_DD	0.0002
	Jun	C33a_DD – C33a_SS, C33a_DS	0.0001, 0.0002
		C33a_DD - C42a_SS, C42a_SD	0.0017, 0.0053
		C33a_SD - C33a_SS, C33a_DS, C42a_SS	0.0001, 0.0001, 0.0041
	Sep	C33a_DD - C33a_SS, C33a_DS	0.009, 0.0014
	C33a_DD	Jun - Mar, Sep, Nov	0.0001
		Mar - Nov, Sep	0.0001, 0.0039
	C33a_SS	Sep - Mar, Nov	0.0045, 0.0077
	C33a_SD	Jun - Mar, Sep	0.0002, 0.0005
		Mar - Nov	0.0088

Stylophora pistillata ($p \leq 0.01$)			
**Season x Symbiont_Treatment**	March	C79_DD - C35a_SS, C78a_SS, C8a_SS, C8a_DD	all 0.0001
		C35a_SS - C35a_SD, C8a_SD	0.001, 0.009
		C35a_SD - C78_SS, C8a_DD, C8a_SS	0.001, 0.003, 0.001
		C8a_SS - C8a_SD	0.001
	June	C79_DD - C79_DS, C35a_SS, C78a_SS	all 0.001
		C79_DD - C8a_SS, C8a_DS	0.001, 0.011
		C79_DS - C35_SD, C78_SD	0.001, 0.006
		C79_DS - C8a_DD, C8a_SD	0.001, 0.004
		C35a_SS - C35a_SD, C8a_DD, C8a_SD	0.002, 0.001, 0.002
		C35a_SD - C8a_SS, C8a_DD	0.002, 0.003
		C78a_SS - C78a_SD, C35a_SD	0.003, 0.003
		C78a_SS - C8a_DD, C8a_SD, C8a_DS	0.002, 0.004, 0.007
		C8a_DD - C8a_SS, C8a_DS	0.001, 0.001
		C8a_SS - C8a_SD, C8a_DS, C78a_SD	0.001, 0.003, 0.001
		C8a_DS - C8a_SD	0.003
	September	C35a_SS - C8a_DD	0.005
		C8a_DD - C8a_SS, C8a_DS	0.007, 0.011
	November	C79_DD - C79_DS	0.001
		C79_DD - C8a_DD, C8a_SD, C8a_DS	0.002, 0.002, 0.004
		C79_DS - C35a_SD, C78_SD	0.003, 0.008
		C79_DS - C8a_SD, C8a_DD	0.001, 0.001
		C35a_SS - C8a_DD, C8a_SD	0.003,0.001
		C35a_SD - C8a_DD, C8a_SD, C8a_DS	0.004, 0.002, 0.004
		C78a_SS - C8a_DD, C8a_SD	0.001, 0.001
		C78a_SD - C8a_SD, C8a_DS	0.005, 0.01
		C8a_DD - C8a_SS, C8a_DS	0.001, 0.001
		C8a_SS - C8a_SD	0.001
		C8a_DS - C8a_SD	0.001
	C79_DD	Nov – Mar, Jun, Sep	0.002, 0.001, 0.007
		June - Mar, Sep	0.001, 0.001
	C35a_SS	Mar - Jun, Sep, Nov	0.005, 0.001, 0.001
		Sep – Jun, Nov	0.009, 0.001
	C35a_SD	Nov – Mar, Jun, Sep	0.004, 0.001, 0.005
		Jun – Mar, Sep	0.001, 0.001
	C78a_SS	Mar - Sep, Nov	0.007, 0.001
	C78a_SD	Jun - Sep, Nov	0.005, 0.003
	C8a_DD	Jun - Sep, Nov	0.001, 0.001
	C8a_DS	Jun - Sep, Nov	0.002, 0.01
	C8a_SS	Mar - Jun, Sep, Nov	all 0.001
		Jun - Nov	0.009
	C8a_SD	Nov – Mar, Jun, Sep	0.003, 0.002, 0.001
		Jun – Mar, Sep	0.001, 0.003

Table S6. Statistics results of 'Symbiont_treatment' for each of the photosynthetic parameters measured in November 2005 (t6).

Nov 2005	Photosynthetic Parameter	df	SS	MS	F	p
Seriatopora hystrix						
	Qm	1	0.010	0.010	0.651	0.430
	F_q'/F_m'	1	0.004	0.004	0.873	0.363
	Dark-adapted F_v/F_m (dusk)	1	0.0003	0.0003	0.197	0.663
Pocillopora damicornis						
	**Qm**	2	0.078	0.039	3.283	**0.049**
	F_q'/F_m'	2	0.033	0.016	2.945	0.066
	Dark-adapted F_v/F_m (dusk)	2	0.0005	0.0003	0.290	0.747
Stylophora pistillata						
	**Qm**	7	0.404	0.058	5.064	**< 0.001**
	$\mathbf{F_q'/F_m'}$	7	0.292	0.042	6.935	**< 0.001**
	**Dark-adapted F_v/F_m (dusk)**	7	0.037	0.005	11.592	**< 0.001**

Table S7. Post-hoc comparisons photosynthetic measurements in November 2005.

Photosynthetic Nov06 (t6)		p
Seriatopora hystrix		
no significant main effects		
Pocillopora damicornis		
**Qm** (MSe = 0.01185, df = 35)	C42a_SD - C33a_DD, C33a_SD	0.0.015, 0.046
Stylophora pistillata	**Between Symbiont_treatments**	
**Qm** (MSe = 0.0114, df = 128)	C8a_DD - C79_DD, C8a_SD, C35a_SD, C78a_SD	<0.001, 0.042, <0.001, 0.005
	C79_DD – C8a_SD, C8a_SS, C35a_SS, C78a_SS	0.002, 0.042, 0016, 0.026
	C8a_SD - C35a_SD	0.048
Fq'/Fm' (MSe = 0.0060, df = 128)	C8a_DD - C79_DD, C8a_SD, C35a_SD, C78a_SD	<0.001, 0.008, < 0.001, <0.001
	C79_DD – C8a_SD, C8a_SS, C35a_SS, C78a_SS	0.004, 0.008, 0.002, 0.021
	C8a_SS –- C35a_SD	0.019
	C35_SS – C35a_SD, C78a_SD, C8a_SD	0.006, 0.046, 0.017
	C35a_SD - C8a_SD, C78a_SS	0.017, 0.032
**Fv/Fm (**MSe = 0.0005, df = 128)	C8a_DD - C79_DD, C8a_SD, C35a_SD, C78a_SD	<0.001
	C79_DD – C8a_SS, C35a_SS, C35a_SD	<0.01
	C8a_SS - C8a_SD, C35a_SD, C78a_SD	≤ 0.001
	C35a_SS - C35a_SD, C78a_SD, C8a_SD	≤ 0.001
	C35a_SD - C8a_SD, C78a_SS	0.002, 0.016
